# Thermo‐Responsive Polymer‐Based Nanoparticles: From Chemical Design to Advanced Applications

**DOI:** 10.1002/marc.202401127

**Published:** 2025-02-02

**Authors:** Giuseppe Nunziata, Marco Nava, Elisa Lacroce, Fabio Pizzetti, Filippo Rossi

**Affiliations:** ^1^ Department of Chemistry Materials and Chemical Engineering “Giulio Natta” Politecnico di Milano via Mancinelli 7 Milano 20131 Italy

**Keywords:** drug delivery, macromolecules, nanoparticles, polymers, thermo‐responsive

## Abstract

Thermo‐responsive polymers have emerged as a cutting‐edge tool in nanomedicine, paving the way for innovative approaches to targeted drug delivery and advanced therapeutic strategies. These “smart” polymers respond to temperature changes, enabling controlled drug release in pathological environments characterized by high temperatures. By exploiting their unique phase transition, occurring at the lower or upper critical solution temperatures (LCST and UCST), these systems ensure localized therapeutic action, minimizing collateral damage to healthy tissues. The integration of these polymers into nanoparticles with hydrophilic shells and hydrophobic cores enhances their stability and biocompatibility. Furthermore, advanced polymer engineering allows precise modulation of LCST and UCST through adjustments in composition and hydrophilic‐lipophilic balance, optimizing their responsiveness for specific applications. In addition to drug delivery, thermo‐responsive nanoparticles are gaining attention in several fields such as gene therapy and imaging. Therefore, this review explores the chemical and structural diversity of thermo‐responsive nanoparticles, emphasizing their ability to encapsulate and release drugs effectively. Second, this review highlights the potential of thermo‐responsive nanoparticles to redefine treatment paradigms, providing a comprehensive understanding of their mechanisms, applications, and future perspectives in biomedical research.

## Graphical abstract

This work explores the chemical design and applications of thermo‐responsive polymer‐based nanoparticles. Focusing on PNIPAM and POEGMA block copolymers, it highlights their thermo‐responsive behaviors and advanced functionalities in imaging, drug delivery, and cell proliferation studies. The study emphasizes the potential of these systems for precise and tuneable advanced applications under temperature‐controlled conditions.

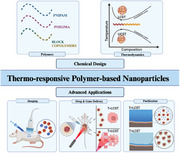

## 1 Introduction

**Figure 1:**
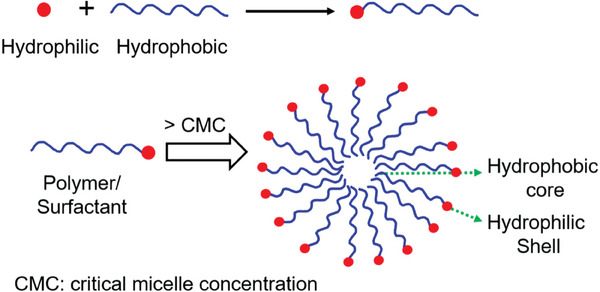
Illustration of the self‐assembly of the polymer into a spherical nanoparticle. Reprinted with permission from.^[^ ^8^ ^]^

Overcoming biological barriers and achieving site‐specific drug delivery remain key challenges in modern medicine.^[^ ^1^ ^]^ Conventional therapies often suffer from limited efficacy due to systemic distribution and off‐target effects, which can lead to severe toxicity in healthy tissues.^[^ ^2^ ^]^ To address these limitations, innovative drug delivery systems capable of responding to specific physiological conditions have garnered increasing attention.^[^ ^3^ ^]^ These systems include organic nanoparticles^[^ ^4^ ^]^ such as liposomes, polymeric nanoparticles, nanogels, micelles, dendrimers, and inorganic nanoparticles^[^ ^5^ ^]^ such as iron oxide, quantum dots, gold nanoparticles, and metal‐organic frameworks. Focusing more on polymeric nanoparticles, these systems are designed using amphiphilic copolymers that form core‐shell structures (**Figure** **1**).^[^ ^6^ ^]^ The hydrophobic core can work as a carrier for hydrophobic drugs,^[^ ^7^ ^]^ while the hydrophilic shell enhances biocompatibility, stability, and potential responsiveness to external stimuli.

**Figure 2:**
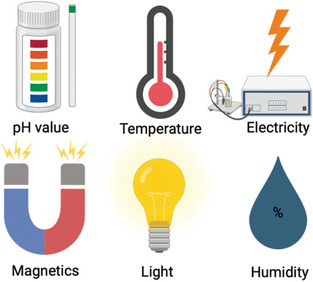
Main types of stimuli for polymeric devices.

This adaptability is further enriched by incorporating “smart polymers”, which are stimuli‐responsive materials capable of undergoing rapid and reversible physical or chemical changes upon exposure to specific environmental triggers.^[^ ^9^ ^]^ These changes, often at the nanoscale, enable precise modulation of the drug release profiles, enhancing therapeutic efficacy and minimizing off‐target effects. Smart polymer systems can respond to a variety of stimuli (**Figure** **2**), which are broadly classified into two categories: physical^[^ ^10^ ^]^ and chemical.^[^ ^11^ ^]^ Physical stimuli include temperature, light, ultrasound, and magnetic or electric fields.

Chemical stimuli involve changes in pH, ionic strength, or the presence of specific biomolecules or chemical agents. Each type of nanoparticle brings unique properties and advantages, making them highly versatile for diagnostics and therapeutics. This versatility positions smart polymeric nanoparticles at the forefront of innovative strategies for overcoming biological barriers and achieving controlled site‐specific drug delivery.^[^ ^12^ ^]^

Particularly, thermo‐responsive polymeric nanoparticles have emerged as a versatile tool for controlled drug release.^[^ ^13^ ^]^ These nanosystems consist of amphiphilic copolymers, where the hydrophobic core serves as a drug reservoir, while the hydrophilic shell imparts stability and ensures interactions with biological fluids. Their temperature‐sensitive behavior allows them to undergo conformational or phase transitions at slightly elevated temperatures, which can be exploited for on‐demand drug release at the target site. Once administered, these nanoparticles traverse the bloodstream and accumulate in specific tissues,^[^ ^14^ ^]^ often aided by phenomena such as the enhanced permeability and retention (EPR) effect.^[^ ^15^ ^]^ The abnormal vascular architecture of diseased tissues, combined with impaired lymphatic drainage, facilitates the selective accumulation and retention of nanoparticles, providing a foundation for their therapeutic action.^[^ ^16^ ^]^ Additionally, the ability of these carriers to maintain drug concentrations within a therapeutic window, above the minimum effective concentration and below the maximum toxic concentration further enhances their clinical potential.^[^ ^17^ ^]^ This review explores the design, mechanisms, and applications of thermo‐responsive polymeric nanoparticles, emphasizing their role in overcoming biological barriers and achieving precise drug delivery. The discussion focuses on their ability to optimize therapeutic outcomes while minimizing side effects, underscoring their transformative impact in the field of advanced drug delivery systems.

This review aims to provide an overview of nanoparticles, with a particular focus on thermo‐responsive ones. Types of NPs and relative applications are presented in the following paragraphs. Thermo‐responsive polymers have emerged as a cutting‐edge tool in nanomedicine, paving the way for innovative approaches to targeted drug delivery and advanced therapeutic strategies. These “smart” polymers respond to temperature changes, enabling controlled drug release in pathological environments characterized by high temperatures. By exploiting their unique phase transition, occurring at the lower or upper critical solution temperatures (LCST and UCST), these systems ensure localized therapeutic action, minimizing collateral damage to healthy tissues. The integration of these polymers into nanoparticles with hydrophilic shells and hydrophobic cores enhances their stability and biocompatibility. Furthermore, advanced polymer engineering allows precise modulation of LCST and UCST through adjustments in composition and hydrophilic‐lipophilic balance, optimizing their responsiveness for specific applications. In addition to drug delivery, thermo‐responsive nanoparticles are gaining attention in several fields such as gene therapy and imaging. Therefore, this review explores the chemical and structural diversity of thermo‐responsive nanoparticles, emphasizing their ability to encapsulate and release drugs effectively. Second, this review highlights the potential of thermo‐responsive nanoparticles to redefine treatment paradigms, providing a comprehensive understanding of their mechanisms, applications, and future perspectives in biomedical research.

This review aims to address gaps in the current literature by offering a unique perspective on thermo‐responsive polymers, including Poly(oligo(ethylene glycol) methyl ether methacrylate) (POEGMA) and Poly(N‐isopropylacrylamide) (PNIPAM). Unlike existing reviews focus on individual polymers,^[^ ^18^, ^19^, ^20^ ^]^ this work places significant emphasis on nanoparticles, exploring novel combinations of hydrophilic thermo‐responsive polymers copolymerized with hydrophobic counterparts to produce amphiphilic copolymers. The review provides an in‐depth discussion of the design, synthesis, and behavior of these nanoparticles, highlighting their potential for a wide range of applications. Furthermore, it provides an analysis of advanced characterization techniques to elucidate the thermo‐responsive behavior of the devices. By integrating these innovative elements, this review seeks to deliver a comprehensive and impactful contribution to the field of thermo‐responsive polymer‐based nanomaterials.

## 2 Thermosensitive Behavior and Mechanism

**Figure 3:**
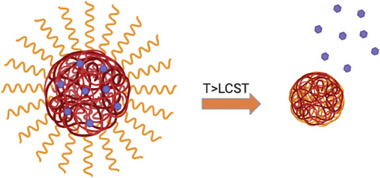
The figure shows the drug release as soon as the temperature exceeds the LCST.

Thermo‐responsive polymeric nanoparticles ensure the stability of NPs in the bloodstream, preventing premature drug release, and allowing drug delivery only in diseased tissues, where temperature variations induce therapeutic release. Specifically, when dealing with thermo‐responsive polymers, two fundamental properties to define are the lower critical solution temperature (LCST) and the upper critical solution temperature (UCST). These properties govern the physical behavior of the polymers, allowing structural changes in response to temperature variations. LCST is defined as the temperature below which a polymer is soluble and above which it becomes insoluble.^[^ ^21^ ^]^ Polymer‐water solution exists as a single phase below the LCST but separates into distinct phases above this temperature. On the other hand, the UCST is defined as the temperature above which the polymer is soluble and below which a phase separation occurs. In contrast to the LCST, phase separation for UCST polymers happens at higher temperatures, with the solution being the single‐phase above the UCST and separating into different phases below it.^[^ ^22^ ^]^ These critical temperatures are essential for the design and application of thermo‐responsive polymers, as they determine the conditions under which the polymers will change their solubility and, consequently, their structural properties. The drug release mechanism relies on exceeding the LCST, allowing for precise control over the timing and rate of drug release in response to temperature changes. This is particularly beneficial for therapies that require targeted delivery.^[^ ^23^ ^]^ The goal is to produce a polymer with an LCST between 38 and 43 °C. By combining their properties, the desired LCST range can be obtained. The following section will provide a detailed overview of the primary types of thermo‐responsive monomers and polymers. When the temperature exceeds the LCST, the thermo‐sensitive shell collapses over the core, resulting in the release of the payload (**Figure** **3**).

**Figure 4:**
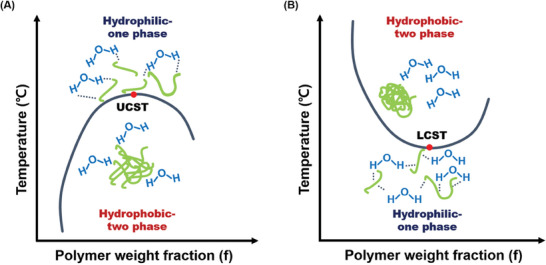
The figure shows the phase behavior of polymers with UCST and LCST transitions, exhibiting the relationship between temperature and polymer weight fraction. A) For UCST polymers, the hydrophilic state (single‐phase) is observed at higher temperatures due to strong hydrogen bonding with water, while the polymer undergoes a phase separation into a hydrophobic two‐phase system as the temperature decreases below the UCST. B) For LCST polymers, the polymer remains hydrophilic and in a single‐phase below the LCST due to favorable hydrogen bonding with water. However, as the temperature increases above the LCST, the polymer transitions into a hydrophobic state, resulting in phase separation (two‐phase system). Reprinted with permission from.^[^ ^27^ ^]^

More in detail LCST and UCST are (**Figure** **4**):

‐LCST: a phase transition occurs from soluble to insoluble upon heating. LCST represents the minimum temperature of the binodal curve of the phase diagram, below which a single phase exists.

Above this temperature, a phase separation into two immiscible phases occurs: a dilute polymer phase and a concentrated polymer phase. This phenomenon is based on the existence of hydrogen bonding between the water molecules and the polymer chain. The polymers with LCST behavior show a sudden and (mostly) reversible change from hydrophilic to hydrophobic behavior that makes them attractive for usage as smart materials.^[^ ^24^ ^]^ Among the water‐soluble thermo‐responsive polymers, those exhibiting LCST are the most common. The thermodynamic basis of this transition can be understood using the Gibbs free energy equation: ΔG = ΔH−TΔS. Below the LCST, the negative enthalpy (ΔH) from hydrogen bonding and the low entropy (ΔS) dominates, making ΔG negative and allowing the polymer to be dissolved. Above the LCST, the endothermic breaking of hydrogen bonds reduces the negative enthalpy contribution, while the gain in ΔS from disrupted water structure becomes the driving force. This results in ΔG variation, leading to phase separation.^[^ ^25^ ^]^

‐UCST: represents the maximum temperature of the binodal curve, above which there is only one phase. In this case, phase separation occurs below the transition temperature. The polymer is miscible with the solvent at higher temperatures, but below the critical temperature, phase separation occurs.^[^ ^26^ ^]^

## 3 Types of Thermo‐Responsive Polymers

Thermo‐responsive polymers can be categorized based on their chemical composition and the mechanisms by which they respond to temperature changes. The most common types are reported in this paragraph.

### 3.1 Poly(N‐Isopropylacrylamide) (PNIPAM)

PNIPAM is one of the most extensively studied thermo‐responsive polymers.^[^ ^28^, ^29^, ^30^, ^31^ ^]^ It is characterized by an LCST of 32 °C,^[^ ^32^ ^]^ making it ideal for biomedical applications, such as drug delivery and tissue engineering. The transition mechanism from a coil‐shaped structure to a compact globular form in aqueous solutions takes place when the temperature surpasses its LCST value. Below the LCST, PNIPAM is soluble in water due to favorable interactions between its amide group and water molecules. As the temperature rises above 32 °C, the interactions between water molecules and the hydrophilic regions of PNIPAM weaken significantly. This weakening allows the hydrophobic isopropyl groups to become more pronounced. Consequently, the polymer undergoes a phase transition, shifting from a hydrated, expanded coil structure to a more compact, globular conformation. This behavior is driven by the thermodynamic favorability of minimizing exposure of the hydrophobic groups to water, leading to a reduction in solubility and resulting in the aggregation of the polymer chains. This transition is crucial for applications such as controlled drug delivery, where the release profile can be precisely manipulated by adjusting the temperature.^[^ ^33^, ^34^ ^]^ Typically, its synthesis is obtained starting from N‐isopropylacrylamide (NIPAM) through free radical emulsion polymerization,^[^ ^35^, ^36^ ^]^ or through reversible‐deactivation radical polymerization (RDRP),^[^ ^37^, ^38^ ^]^ and conjugation chemistries like “click chemistry”.^[^ ^39^, ^40^ ^]^ These approaches enable precise control over macromolecular architecture and facilitate functionalization with biomolecules and nanoparticles. The LCST can be tuned by changing several factors, including the molecular structure of the polymer and the aqueous environment in which it is used. The molecular structure of PNIPAM plays a significant role in determining its properties by affecting the balance between hydrophilicity and hydrophobicity. Modifications to the polymer, such as end‐groups and side chains that increase hydrophilicity, tend to raise the lower critical solution temperature (LCST). Furyk and coworkers studied the influence of various end groups on the LCST of PNIPAM and demonstrated that the LCST of trityl‐containing PNIPAM fractions varied as the molecular weight decreased below 100 kDa.^[^ ^41^ ^]^ Conversely, modifications that enhance hydrophobicity lower the LCST.

Additionally, the tacticity and sequence distribution of the polymer are important factors. For example, the random or block incorporation of isotactic or syndiotactic sequences can change the solubility of PNIPAM, which in turn affects its LCST.^[^ ^42^ ^]^ Second, the aqueous environment surrounding PNIPAM also affects its LCST. The presence of salts, described by the Hofmeister series, can either stabilize or destabilize the polymer's interaction with water. More specifically, kosmotropic salts and ammonium sulphate promote water structure around the polymer, raising the LCST by enhancing hydrophobic interactions.^[^ ^43^ ^]^ On the other hand, chaotropic salts and sodium thiocyanate salts, disrupt water structure, lowering the LCST by decreasing the solubility of the polymer. Then, the addition of organic solvents to the aqueous environment can influence the LCST through the co‐nonsolvent effect.^[^ ^44^ ^]^ Small amounts of good organic solvents, such as methanol, can decrease the LCST, while higher concentrations might increase it. This is due to the complex interactions between the polymer, water, and the organic solvent, which can alter the solubility profile of the polymer. Constantin and coworkers developed dexamethasone‐loaded PLGA NPs with a shell of PNIPAM and 4‐vinyl pyridine (PVP) produced via electrostatic interactions.^[^ ^45^ ^]^ This shell led thermo‐responsive behaviour in the NPs. Specifically, below the LCST, the PVP component formed a hydrogel barrier around the NPs surface, which slowed the diffusion of the drug. Above the LCST (37 °C), the PVP hydrogel became hydrophobic and shrank, further restricting drug diffusion. As a result, the NPs prepared in this study demonstrated a promising alternative to traditional Poly Lactic‐co‐Glycolic Acid (PLGA) NPs, offering controlled release properties influenced by temperature.

### 3.2 Poly(Ethylene Glycol) (PEG) Based Polymers

Since its discovery, PEG has been extensively employed, particularly for its ability to elude immune recognition and prevent opsonization when applied to nanoparticles.^[^ ^46^, ^47^ ^]^ Polyethylene glycol (PEG) is characterized by its molecular structure (‐CH₂‐CH₂‐O‐)_n_, where oxygen atoms form multiple hydrogen bonds. This structural feature allows PEG chains to attract water molecules in aqueous environments, forming a protective hydration layer on nanoparticle surfaces. This hydrated “pillow” effectively repels opsonin,^[^ ^48^ ^]^ preventing their binding and giving nanoparticles stealth properties that protect them from phagocytic cells.^[^ ^49^ ^]^ Hence, PEG is hailed as the standard for mitigating opsonization. Furthermore, PEG chemical versatility enables the function of its terminal ends with various groups, enhancing its applicability in biomedicine. PEGylation of nanoparticles increases their stability under physiological conditions,^[^ ^50^ ^]^ promotes long circulation in the bloodstream,^[^ ^51^ ^]^ and confers them thermo‐responsive activities.^[^ ^52^, ^53^ ^]^

PEG‐based polymers can bring about to exhibit thermo‐responsive properties by including them with other monomers. These polymers are valued for their special biocompatibility and find extensive use in advanced medical applications, including drug delivery systems and tissue engineering scaffolds. Moreover, PEGylation influences the Z‐potential of nanoparticle suspensions. At acidic pH levels ≈4–5, nanoparticles exhibit enhanced colloidal stability due to the negative charge provided by dissociated hydroxyl groups.^[^ ^54^ ^]^ However, at physiological pH, the Z‐potential nears zero, leading to potential particle precipitation. So, PEGylation also increases protein half‐life by impeding enzymatic degradation, causing a reduction of the cytotoxicity in PEGylated nanoparticles, making them high suitable for biomedical applications. Over the years, its structure has been modified to form more complex architectures that possess thermo‐responsive properties themselves, as seen in the case of poly(oligo(ethylene glycol) methacrylate).

### 3.3 Poly(Oligo(Ethylene Glycol) Methacrylate) (POEGMA)

Since the 1990s, poly(oligo(ethylene glycol) methyl ether methacrylate) (POEGMA) has reaped increasing interest following the development of controlled synthesis techniques.^[^ ^55^, ^56^, ^57^ ^]^ The structure of POEGMA consists of a methacrylate polymer backbone with oligoethylene glycol (OEG) groups attached to each monomer, which confer the thermo‐responsive behavior. When the temperature exceeds the LCST, the hydrophilic OEG groups tend to “self‐associate” and exclude water, resulting in a decrease in the polymer's solubility in aqueous environments.^[^ ^58^ ^]^ Starting from different monomers it is possible to obtain a wide range of polymer classes including variants like POEGMA_2_ and POEGMA_8_. They possess LCST values ranging from 26 to 90 °C,^[^ ^59^ ^]^ depending on the different ethylene glycol chain lengths. LCST can be adapted by adjusting the chain length and composition, allowing for precise thermal proprietaries control. POEGMA_n_ are valued for their biocompatibility and are used in a range of biomedical and industrial applications.^[^ ^60^ ^]^ Lutz and coworkers demonstrated that, by statistically copolymerizing two or more specific monomers, it is feasible to achieve any LCST within the range defined by the homopolymers of those monomers, while maintaining the copolymer's uniformity.^[^ ^61^ ^]^ Furthermore, it has been discovered that the LCST has a linear proportion with the composition of the monomers within the polymer. This represents an important advancement in the field of POEGMA's utilization: indeed, by selecting the quantity of the monomer it is possible to fine‐tune the transition temperature to the desirable value.

Moreover, this type of polymer is able to overcome a substantial matter associated with the thermal behavior of the PNIPAM, in which it has been reported a strong difference between different cycles of cooling and heating. This problem is referred to as hysteresis. This represents a significant advantage, as the molecule can be used without concerns about thermal behavior, regardless of the temperature cycles it undergoes leading to uniform and reproducible features.^[^ ^62^ ^]^ With the advent of modern controlled polymerization techniques, as well as the commercial availability of various EGMA monomers, it has become straightforward to produce well‐defined homo‐ and copolymers of POEGMA. Polymerization can occur in aqueous media, simplifying combination with biomaterials.^[^ ^63^ ^]^ POEGMA can also be directly synthesized from initiator‐functionalized (bio)macromolecules and solid substrates, enabling the creation of bio‐hybrid materials for a wide range of applications.^[^ ^64^ ^]^ An illustrative application of POEGMA‐based polymers in thermo‐responsive systems involves dendronized nanoparticles in the work of Zhang and coworkers.^[^ ^65^ ^]^ These NPs, loaded with Donor‐Acceptor Stenhouse Adducts (DASAs), exhibit temperature‐dependent behaviour due to the interactions between the hydrophobic DASA moieties and dendritic OEG groups. DASAs are thermo‐responsive organic molecules that undergo reversible structural changes in response to heat stimuli. Their hydrophobicity and ability to interact with polymer matrices make them ideal for enhancing the functionality of responsive systems. The thermo‐responsive behaviour of these systems is characterized by a reduction in cloud point temperature (Tcp) upon increasing DASA concentrations. The incorporation of hydrophobic dyes significantly enhances the entropy‐driven thermal dehydration of dendritic POEGMAs, further reducing Tcp. These nanoparticles demonstrate the versatility of OEG‐based polymers, offering a promising platform for the development of stimuli‐responsive nanocarriers in drug delivery and diagnostics.

## 4 Types of Thermo‐Responsive Co‐Polymers

Block copolymers, composed of distinct segments of different polymers, can exhibit exclusive thermo‐responsive activities, making them extremely versatile in various applications. By integrating a thermo‐responsive block with either a hydrophilic or a hydrophobic block, these polymers can form micelles or other nanostructures being able to change their proprieties and conformation when subjected to temperature changes. This transformation occurs due to the different solubility of the blocks, causing self‐assembly or disassembly in response to heat. The reason this process occurs lies in thermodynamics: it is governed by an unfavorable mixing enthalpy combined with a low mixing entropy.^[^ ^66^ ^]^

Once self‐assembly has occurred, the composition, the number of repeating units, and the Flory‐Huggins interaction parameter are the main factors that influence the morphology of the structure.^[^ ^67^ ^]^ These factors determine the formation of various structures, including spheres, cylinders, gyroids, and lamellae.^[^ ^68^, ^69^ ^]^ By carefully tuning these parameters, it is possible to control the self‐assembly process to achieve the desired nanostructures. The self‐assembly behavior of block copolymers can also be influenced by external parameters such as mechanical or electric fields.^[^ ^70^, ^71^ ^]^ In particular, amphiphilic polymers can spontaneously organize themselves, in aqueous conditions, into order structures strongly influenced by the architecture of the monomer.^[^ ^72^ ^]^ Additionally, the environment in which they are located influences the process; the main external factors are the temperature, ion strength, pH, and the concentration of the monomer.^[^ ^73^ ^]^ To better understand the self‐assembly phenomena, it is required to study the main thermodynamic force responsible for initiating the process. It has been proved that the main contribution is associated with the dehydration and the subsequent collapse of the hydrophobic portion. Additionally, electrostatic interaction and intramolecular forces, such as hydrogen bond, enhance the stability and favor the self‐assembled structure. The self‐assembly process is significantly affected by electrostatic interactions within the copolymer blocks, promoting the formation of well‐defined nanostructures.^[^ ^74^ ^]^ Self‐assembly is a fundamental process in drug delivery systems, particularly for encapsulating therapeutic agents within nanoparticles. This can be achieved through methods such as nano‐ precipitation^[^ ^75^ ^]^ or emulsion‐evaporation techniques,^[^ ^76^ ^]^ both of which facilitate the formation of stable polymeric nanoparticles capable of carrying drugs effectively.^[^ ^77^ ^]^

### 4.1 PNIPAM–b‐PLA

Incorporating various types of monomers, it can be possible to get a material with diverse properties, such as biodegradability, amphiphilic behavior, self‐assembly feature and stable structures. For instance, this can be achieved by adding polyester polylactic acid (PLA), which can be synthesized through ring‐opening polymerization (ROP).^[^ ^78^, ^79^ ^]^ This method avoids any thermodynamic limitations of polycondensation reactions that produce water molecules,^[^ ^80^ ^]^ thus allowing higher conversion rates and the production of high molecular weight polymers. It is possible to create amphiphilic particles where the hydrophobic core is composed of PLA, capable of encapsulating hydrophobic drugs.^[^ ^81^ ^]^ The outer shell, meanwhile, is made of thermo‐responsive polymers such as PNIPAM which, according to literature, has been formed through RAFT polymerization.^[^ ^82^, ^83^ ^]^

This dual‐structure configuration offers significant advantages for drug delivery applications: PLA is known for its excellent biodegradability and biocompatibility.^[^ ^84^ ^]^ In biomedical applications, this ensures that the carrier breaks down into non‐toxic lactic acid within the body. This degradation process prevents any toxicity associated with long‐term accumulation, as the substance is ultimately eliminated through glomerular filtration.^[^ ^85^, ^86^ ^]^ The degradation process can be easily controlled by adjusting the molecular weight: the higher the molecular weight, the longer the degradation process. Also, the crystallinity of PLA affects this process. In this way, by adjusting those parameters, it is possible to design a desired life‐time degradation that matches with specific therapeutic requirements. The LCST of the material has been found to be slightly higher than that of pure PNIPAM.^[^ ^87^ ^]^ The reason is attributed to the influence of hydrophilic hydroxyl groups on this thermal transition: in fact, as the literature reported, it is well‐established that the incorporation or grafting of PNIPAM with hydrophobic polymers can modify the LCST, thereby improving its compatibility with more hydrophobic environments.^[^ ^88^, ^89^, ^90^ ^]^ Moreover, as the hydrophobicity and polydispersity of the hydrophobic blocks increase, the LCST may become less distinct. This phenomenon can lead to a less precise cargo release temperature for the micelles.^[^ ^91^ ^]^

### 4.2 PEG‐b‐PCL

To optimize the design of nanoparticles for biomedical applications, one effective strategy involves the synthesis of a copolymer that combines the beneficial properties of different polymers: a promising approach is to create a copolymer composed of poly‐ε‐caprolactone (PCL) and PEG. PCL is a well‐established biocompatible and biodegradable polyester commonly used in medical sutures. Its key advantages include its biocompatibility, which ensures that it can be safely used in the body, and its biodegradability, which allows for the gradual collapse and elimination of the material without causing long‐term accumulation.^[^ ^92^ ^]^ However, PCL is also hydrophobic, which can lead to interactions with proteins and confer the ability to encapsulate drugs. Furthermore, synthesized core–shell nanoparticles, with thermo‐responsive properties by grafting POEGMA, constitute the hydrophilic component and PCL onto a polymer backbone. POEGMA serves as the thermo‐responsive block, while PCL represents the lipophilic block, resulting in smart core–shell nanoparticles.^[^ ^53^ ^]^ In order to obtain a precise transition temperature, one method involves the selection of a precise composition of the monomers to tailor the LCST of the block copolymer, setting it slightly above 37 °C. Beyond this aspect, there is the ratio between the hydrophobic and hydrophilic segments.

As usual, it has been reported that if the hydrophobic PCL is shorted than the hydrophilic portion, the polymer presents an enhanced tendency to aggregate after heating. Hence, it is possible to underline the strong influence of the hydrophilic component over the thermal response behavior.^[^ ^53^ ^]^ The discussion so far is closely tied to the concept of Hydrophilic‐Lipophilic Balance (HLB). The ability of the copolymer to undergo significant temperature‐induced aggregation and the effectiveness of its thermo‐responsive behavior are intricately linked to the HLB value: above the LCST, higher HLB values correlate with increased levels of aggregation. This is because a higher HLB indicates a greater proportion of hydrophilic content relative to the hydrophobic components in the copolymer. Consequently, copolymers with elevated HLB values exhibit more pronounced aggregation behavior when subjected to temperature changes, as their hydrophilic segments promote stronger intermolecular interactions and aggregation. Specifically, substantial aggregation occurs when the HLB value exceeds 11, with aggregation percentages above 50%. Conversely, when the HLB value is lower than 10, the extent of aggregation is significantly reduced, with an extension of aggregation under 20%.^[^ ^53^ ^]^

### 4.3 Pluronic (PEG‐PPG‐PEG)

One notable example of a thermo‐responsive block copolymer is Pluronic, also known as poloxamer. Pluronic is a tri‐block copolymer consisting of alternating PEG and polypropylene glycol (PPG) segments. Its structure comprises two hydrophilic PEG blocks flanking a central hydrophobic PPG block. At a temperature below the critical temperature (20–25 °C), Pluronic behaves like PEG and remains soluble in water.^[^ ^93^ ^]^ As the temperature rises above this threshold, the PPG block undergoes a phase transition, leading to the formation of micelles. When the concentration of these micelles reaches a sufficient level, they can aggregate to form hydrogels.^[^ ^94^ ^]^ Pluronic's unique temperature‐sensitive behavior enables it to switch from a soluble state to a gel‐like state upon heating. This property is leveraged in various biomedical and industrial applications. Notably, Pluronic has been found to inhibit P‐glycoprotein,^[^ ^95^ ^]^ which plays a role in drug resistance. Pluronic's versatility is further demonstrated by its availability in different molecular weights and various ratios of hydrophilic to hydrophobic blocks, making it suitable for a range of applications. Its biocompatibility, owing to the PEG segments, combined with its temperature‐responsive behavior, makes it a valuable material.

### 4.4 N‐Substituted Acrylamide

These polymers included a wide range of polymers but they have not gained as much attention as PNIPAM.^[^ ^96^ ^]^ The thermo‐sensitivity and solution performance of these polymers are significantly influenced by the structure of the acrylamide's alkyl group.^[^ ^97^ ^]^ To achieve N‐substituted polyacrylamides with enhanced properties and specific LCST, a detailed understanding of their behavior is essential. To realize this, a series of these polymers was synthesized by varying the ratio of hydrophobic to hydrophilic components; that study figures out the influence of the composition over the LCST and describes how the HLB ratio alters its value. These polymers were tested for their solubility across different temperatures, with some being completely insoluble in water and others fully soluble at all temperatures allowing the determination of the LCST. The copolymers studied included poly(DEA‐co‐AA), poly(DEA‐co‐DMA), poly(DEA‐co‐EA), poly(TBA‐co‐EA), and poly(TBA‐co‐DMA), listed according to their hydrophilic properties. In this experiment, it was valuated the sequence of hydrophilicity among the N‐substituted acrylamide monomers, confirming the actual and hypothetical LCST values.^[^ ^98^ ^]^ So, the LCST can be modified according to the composition of the comonomers by varying the hydrophilicity/hydrophobicity ratio. At lower temperatures, the dissolution of the polymer is promoted by hydrogen bonds formed between the hydrophilic amide groups and water. As the temperature increases, these hydrogen bonds start to break down, and hydrophobic interactions among the polymer chains take precedence, ultimately resulting in phase separation.

### 4.5 Copolymers of PNVCL

In both theoretical and experimental studies, copolymerization of N‐vinylcaprolactam (PNVCL) with various monomers has been utilized to introduce desired functionalities while maintaining its temperature‐responsive properties.^[^ ^99^ ^]^ By incorporating ionic, hydrophilic, or hydrophobic functional groups, the properties of PNVCL can be tailored without compromising its core temperature‐responsive behavior. The addition of a second monomer, typically more hydrophilic, results in an increased phase separation temperature in aqueous solutions. This effect is gradual initially and becomes more pronounced when the second monomer exceeds 40–50 mol% of the total monomer content. For instance, the inclusion of N‐vinylpyrrolidone (NVP) in PNVCL copolymers has been shown to raise the lower critical solution temperature (LCST) from 33 to 80 °C with increasing NVP content. Similarly, the introduction of N‐vinyl‐N‐acetamide (VMA) also increases the LCST.^[^ ^99^ ^]^

Copolymers of PNVCL with ionic comonomers such as acrylic acid (AA),^[^ ^100^ ^]^ methacrylic acid (MAA),^[^ ^101^ ^]^ 2‐methacryloyloxybenzoic acid (2MBA), maleic acid (MA),^[^ ^102^ ^]^ and 5‐vinyltetrazole (VT),^[^ ^103^, ^104^ ^]^ exhibit enhanced complexation abilities compared to their homopolymers. These copolymers are pH and temperature‐sensitive, with phase separation temperature being influenced by the pH of the medium, thus broadening the range of practical applications for NVCL‐based polymers.^[^ ^99^ ^]^

### 4.6 Poly(2‐(Dimethylamino)Ethyl Methacrylate) (PDMAEMA)

Poly(2‐(dimethylamino)ethyl methacrylate) (PDMAEMA) exhibits volume phase transition determined by hydrogen bonding interactions. This transition occurs when it becomes more thermodynamically favorable for the system to separate into different phases by breaking the hydrogen bonds between water molecules and the polymer chains. As an outcome, the polymer film undergoes dehydration and collapses above its LCST, which strictly depends on the pH.

It has been demonstrated that the temperature‐responsive properties of PDMAEMA predominantly explore its comportment in relation to pH changes, particularly when used in polymer brushes attached to nanoparticles. For instance, Zhang and coworkers observed that PDMAEMA brushes attached to nanoparticles showed minimal temperature responsiveness at pH 3, where the polymer brush remains in a fully ionized state.^[^ ^105^ ^]^ On the other hand, increasing the level of pH up to 10, a marked level of LCST, about 31 °C, has been found accompanied by a change in optical transmittance. Also, even at pH 8, the thermo‐responsive behavior was less pronounced, though still evident.^[^ ^106^ ^]^ Moreover, supporting this observation, another study investigated PDMAEMA grafted onto silica nanoparticles and found that the LCST was significantly affected by the pH level. In particular, the study revealed that higher pH values resulted in a decrease in the LCST, with temperatures ranging from 20 to 68 °C for pH levels of 6, 7, and 8, respectively.^[^ ^107^ ^]^ Additionally, the research demonstrated that increasing the molecular weight of PDMAEMA at a fixed pH led to a lower LCST; for example, at pH 6, the LCST decreased from 53 to 48 °C as the molecular weight was increased.^[^ ^107^ ^]^ Even after collapsing above the LCST, PDMAEMA brushes hold a degree of hydration.^[^ ^108^ ^]^ This residual hydration is apparent from two observations: first, the water content in the collapsed brushes remains ≈40%, and second, the refractive indices of the collapsed brushes indicate the presence of water within the system (the refractive indices of the brushes in their ambient dry state are higher). This phenomenon is explained by the high grafting density of the brushes, which prevents them from collapsing completely due to significant entropic costs.^[^ ^108^, ^109^ ^]^ Additionally, ionizable polymers like PDMAEMA exhibit strong hydration even at low grafting densities. The partial charge on the polymer brush also contributes to its hydration behavior.

## 5 Characterization of Thermo‐Responsive Properties

Understanding the behavior of thermo‐responsive polymers under diverse environmental conditions is crucial for optimizing their performance in specific applications. This paragraph delves into the methodologies employed to scrutinize the physical, chemical, and mechanical properties of these polymers. Through meticulous characterization, insights into their phase behavior, thermal stability, responsiveness to temperature fluctuations and interactions with different substances are systematically explored and elucidated. The study utilizes a blend of experimental techniques and computational modelling to deepen the comprehension of thermo‐responsive polymer systems. Among the various types of analysis, there is the presence of Fourier transform infrared spectroscopy (FTIS), dynamic light scattering (DLS), nuclear magnetic resonance (H‐NMR), scanning electron microscopy (SEM), atomic force microscopy (AFM), X‐ray diffraction analysis (XRD) and UV–vis spectroscopy (UV). FTIR spectroscopy can be employed to determine the LCST of a polymer,^[^ ^110^, ^111^ ^]^ the swelling behavior^[^ ^112^ ^]^ and to study hydrogen bonding within polymers.^[^ ^113^ ^]^ For this purpose, the temperature is increased within a range, for example from 22 to 47 °C, with a spectrum recorded at each degree Celsius using an effective heating rate of ≈0.07 °C per min. A low heating rate is chosen for both techniques to ensure thermal equilibration and to prevent an artificial shift in the LCST due to rapid heating.^[^ ^114^ ^]^ FTIR spectroscopy proves to be a valuable tool for determining the LCST of polymers and gels. The shift in the maxima of the asymmetric C‐H stretching bands as a function of temperature provides a reliable measure of the LCST. Below the LCST, most of the carbonyl groups of the amide groups are involved in hydrogen bonding with water. Above the LCST, ≈20% of these bonds are replaced by intramolecular bonds. Additionally, results suggest that both the side groups and the backbone of the polymer undergo conformational changes during the transition. In addition, DLS measurement can be used to measure the thermo‐responsive properties of a sample. Specifically, a temperature ramp can be employed to observe the changes in particle size as a function of temperature to study temperature‐induced phase separation in aqueous solutions.^[^ ^115^ ^]^ Nuclear Magnetic Resonance (NMR) spectroscopy is a powerful analytical technique based on the absorption of electromagnetic radiation in the radio frequency range by atomic nuclei.

It is possible to use H‐NMR in temperature to discover the mechanism of the phase transition due to the temperature and to find out the value at which this change occurs. Taking the polymer HBPO‐PNIPAM as an example,^[^ ^116^ ^]^ this phenomenon is primarily driven by the secondary aggregation of micelles, which occurs due to increasing hydrophobic interactions resulting from the dehydration of PNIPAM shells upon heating.^[^ ^117^ ^]^ To delve into the molecular mechanisms underlying the LCST transition, a variable temperature H‐NMR analysis was conducted. Usually, it is added an inert compound to take as a reference, such as potassium hydrogen which maintains its signal at ≈7.5 ppm with consistent intensity across different temperatures.^[^ ^118^ ^]^ SEM instruments can achieve extremely high resolutions, often better than 1 nano‐meter, making them crucial for studying a wide range of materials at the microscopic level.^[^ ^119^ ^]^ Regarding SEM imaging under controlled temperature conditions, the process begins with preparing samples by soaking them in water to achieve complete swelling at temperature es ranging from above to below the LCST.^[^ ^111^ ^]^ Once swollen, the samples are quickly frozen using liquid nitrogen to preserve their hydrogel structure. Subsequently, they undergo dehydration using a freeze dryer over around two days to completely remove water content.^[^ ^120^ ^]^ Temperature sensitivity is evaluated based on changes in the gap of lyophilized hydrogels. Thermo‐responsive hydrogels exhibit dynamic changes in conformation following the cooling treatment. Notably, the pore sizes of these gels can increase significantly compared to their original dimensions at higher temperatures.^[^ ^121^ ^]^ The interaction between ultraviolet radiation and molecules forms the basis of UV–vis spectroscopy. UV–vis can also be used to analyze thermo‐responsive properties:^[^ ^122^ ^]^ indeed, the temperature can be precisely controlled in a fixed temperature range, for example from 10 to 60 °C using the spectrometer's temperature control unit. In order to investigate if PNIPAM/PSt^[^ ^123^ ^]^ micelles exhibit a thermal response comparable to the well‐documented behavior of the PNIPAM homopolymer, it was analyzed the optical transmittance of an aqueous solution of these polymeric micelles as a function of temperature.^[^ ^83^ ^]^ The findings revealed a distinctive and significant characteristic of the PIPAM/PSt micelles; as depicted in the next figure, these micelles undergo a structural transformation at a temperature corresponding to the LCST of PNIPAM, ≈32 °C. Atomic force microscopy (AFM) can also be used to study the thermo‐responsive of polymers: indeed, the temperature of the sample cell was controlled at 25 or 40 °C using a warm plate, with the temperature monitored by a digital thermometer equipped with a thermocouple. Force measurements were obtained by multiplying the cantilever deflection by its spring constant and were plotted against the cantilever's z‐displacement.^[^ ^124^ ^]^

To ensure consistency and minimize the uncertainty in force determination, the same cantilevers were used throughout a series of measurements on PNIPAM surfaces at different temperatures. The following results highlight the significant impact of temperature on the structural properties of the PNIPAM graft layers. At 25 °C, the swollen thickness and repulsive thickness of the polymer layer were ≈1.8 times and 1.2 to 2.8 times greater than the compression thickness, respectively. This suggests that the polymer layer was more swollen and exhibited greater repulsive thickness at this lower temperature. In contrast, at 40 °C, the swollen thickness was similar to the compression thickness, and the repulsive thickness was minimal, less than 10 nm. This indicates that at 40 °C (above the LCST), the PNIPAM graft layer underwent substantial collapse, with the formation of a much tighter structure and the absence of a significant diffuse layer. These observations underscore the crucial role of temperature in modulating the structural behavior of PNIPAM graft layers, influencing their swelling and repulsive properties.^[^ ^124^ ^]^ Moreover, the AFM analysis of thermo‐responsive films made from PNIPAM indicates that properties such as Young's modulus, film thickness, and surface roughness are affected by the polymer's phase transition.^[^ ^125^ ^]^ As the temperature approaches the LCST, the polymer chains undergo cooperative hydration, transitioning from a collapsed state to an expanded coil. This results in significant swelling and a marked softening of the film within a narrow temperature range. Conversely, when the temperature exceeds the LCST, the polymer chains experience cooperative dehydration, leading to film shrinkage and increased stiffness.^[^ ^126^ ^]^

## 6 Applications

Thermo‐responsive polymers have developed decisive applications in a wide range of fields, including biomedical areas such as drug delivery and tissue engineering, as well as catalysis,^[^ ^127^ ^]^ purification,^[^ ^128^, ^129^ ^]^ and additive manufacturing, like 3D printing and fabrication of complex structures.^[^ ^130^ ^]^ Additionally, polymeric materials are extensively used in the pharmaceutical field, mainly because smart polymers can preserve the composition and structure of biological substances such as genes, proteins, cells, and drugs, forming a stable complex for their duration of use.^[^ ^131^ ^]^ Among smart polymers, nanoparticles are the most notable thermo‐responsive structures. Thermo‐responsive nanoparticles with LCST are widely employed as depots for tissue regeneration or for sustained and localized drug delivery.^[^ ^132^ ^]^ In environmental science, thermo‐responsive polymers are leveraged for water purification technologies.

Their ability to undergo phase transitions in response to temperature changes allows for the effective elimination of pollutants and contaminants, making water clean.^[^ ^133^ ^]^

### 6.1 Drug Delivery

Drug delivery is a prominent application of thermo‐responsive polymers, leveraging their unique temperature‐sensitive properties to control the release of therapeutic agents. This approach capitalizes on the ability of these polymers to undergo reversible phase transitions in response to temperature changes, thereby enabling precise modulation of drug release rates.

**Figure 5:**
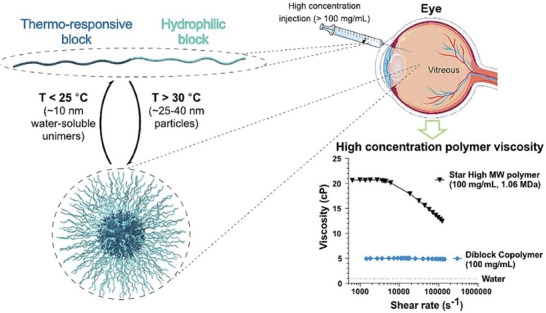
Representative image of thermoresponsive particles developed for ocular injection, illustrating their size with temperature changes and the corresponding viscosity profile critical for delivery and retention. Reprinted with permission from.^[^ ^134^ ^]^

By integrating such polymers into drug delivery systems, it is possible to achieve targeted and controlled release profiles that enhance therapeutic efficacy and minimize side effects. Fergie and coworkers developed a novel amphiphilic diblock polymer composed of NIPAM as a thermo‐responsive block for intravitreal drug delivery (**Figure** **5**).^[^ ^134^ ^]^ This copolymer addressed critical limitations of current drug delivery platforms for volume‐restricted tissues, achieved through precise particle size control using thermo‐responsive amphiphilic diblock copolymers. Its tailored design not only enhances delivery efficiency but also demonstrates promising potential for applications requiring suitability for ocular injections.

**Figure 6:**
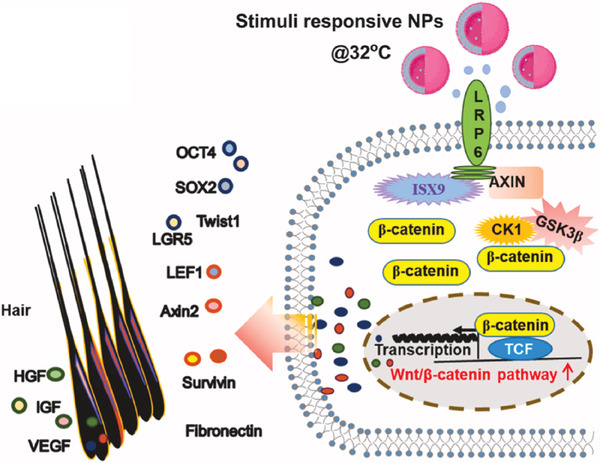
A schematic representation of the action of ISX9@TBNPs for hair regeneration via the Wnt/β‐catenin pathway. ISX9 beleaguered Axin1 and strengthened the LRP6‐Axin1 interaction, in this manner causing the stabilization of β‐catenin and raised up expression of Wnt, hair and stemmness marker genes for hair regeneration. Reprinted with permission from.^[^ ^138^ ^]^

In contrast, in another study, a thermo‐responsive magnetic nanoparticle coated with poly(N‐isopropylacrylamide)/N‐(hydroxymethyl)acrylamide was developed. This hybrid system was designed to combine hyperthermia with the controlled release of doxorubicin, aiming to enhance its synergistic anticancer efficacy, as demonstrated through in vitro testing.^[^ ^135^ ^]^ Thermo‐responsive nanoparticles can be exploited also for cancer drug delivery. There was developed a formulation of curcumin with biodegradable thermoresponsive chitosan‐*g*‐poly (*N*‐vinylcaprolactam) nanoparticles for targeted cancer drug delivery. Curcumin, a bioactive compound with anticancer properties, was encapsulated in spherical nanoparticles (≈220 nm), which demonstrated high loading efficiency and stability. Biological evaluations confirmed the selective release of curcumin at temperatures above the lower critical solution temperature (LCST), aligning with the tumor microenvironment's slightly elevated temperatures. Cellular studies showed that the nanoparticles exhibited specific cytotoxicity to cancer cells at temperatures above the LCST, with minimal toxicity to normal cells across a range of concentrations. Mechanistic studies revealed that apoptosis was mitochondrial‐mediated, as confirmed by JC‐1 assays, and flow cytometry indicated increased apoptosis in cancer cells compared to normal cells.^[^ ^136^ ^]^ Another advancement in drug delivery systems is the dual‐targeting strategy for methotrexate, which holds significant promise for improving cancer therapy, particularly for tumors overexpressing CD44 receptors. This approach leverages two key tumor characteristics: the elevated temperature of tumor tissues and the overexpression of CD44, integrating these features into a single drug delivery system. The use of thermosensitive nanoparticles combined with a CD44‐targeting ligand ensures that the nanostructures exhibit selectivity, delivering the therapeutic payload predominantly to cancer cells while sparing healthy tissues.^[^ ^137^ ^]^ Nonetheless, tumor heterogeneity poses challenges, such as variability in CD44 expression, tumor size, and local hyperthermic conditions, which could affect the efficacy of this system. The optimization of nanoparticle composition and processing parameters is therefore crucial to achieving consistent performance. Such dual‐targeting strategies not only enhance drug efficacy and reduce off‐target effects but also pave the way for adapting similar approaches to intracellular targets, such as those involved in mitochondrial biogenesis. By combining an understanding of tumor biology, sophisticated chemical design, and advanced formulation techniques, these innovations hold great promise for improving the safety and effectiveness of cancer chemotherapies. A significant example is the development of chitosan‐based nanocarriers functionalized with poly(glycidyl methacrylate‐co‐N‐isopropyl acrylamide) and stabilized by a PF127 core (**Figure** **6**).^[^ ^138^ ^]^

This system, designed for the delivery of ISX9, a neurogenesis inducer, has demonstrated remarkable efficacy in the treatment of androgenetic alopecia. Through precise temperature sensitivity, these nanoparticles release the drug at skin temperature, ensuring a sustained and localized effect. This feature allowed the ISX9‐loaded nanoparticles to outperform conventional topical ISX9 applications by significantly enhancing the expression of genes involved in the Wnt/β‐catenin signaling pathway, a key regulator of hair follicle regeneration. Additionally, the system was shown to induce higher levels of stem cell and hair‐related markers, including VEGF, IGF‐1, and LGR5, highlighting its potential in promoting hair regrowth. Moreover, the in vivo safety profile of these nanoparticles was rigorously assessed through histological examination of major organs, with no signs of toxicity observed, further supporting their clinical applicability.

These findings suggest that thermo‐responsive nanoparticles not only offer an innovative platform for the efficient delivery of hydrophobic drugs but also provide a new avenue for treating conditions such as androgenetic alopecia with greater precision and efficacy.

### 6.2 Purification

**Figure 7:**
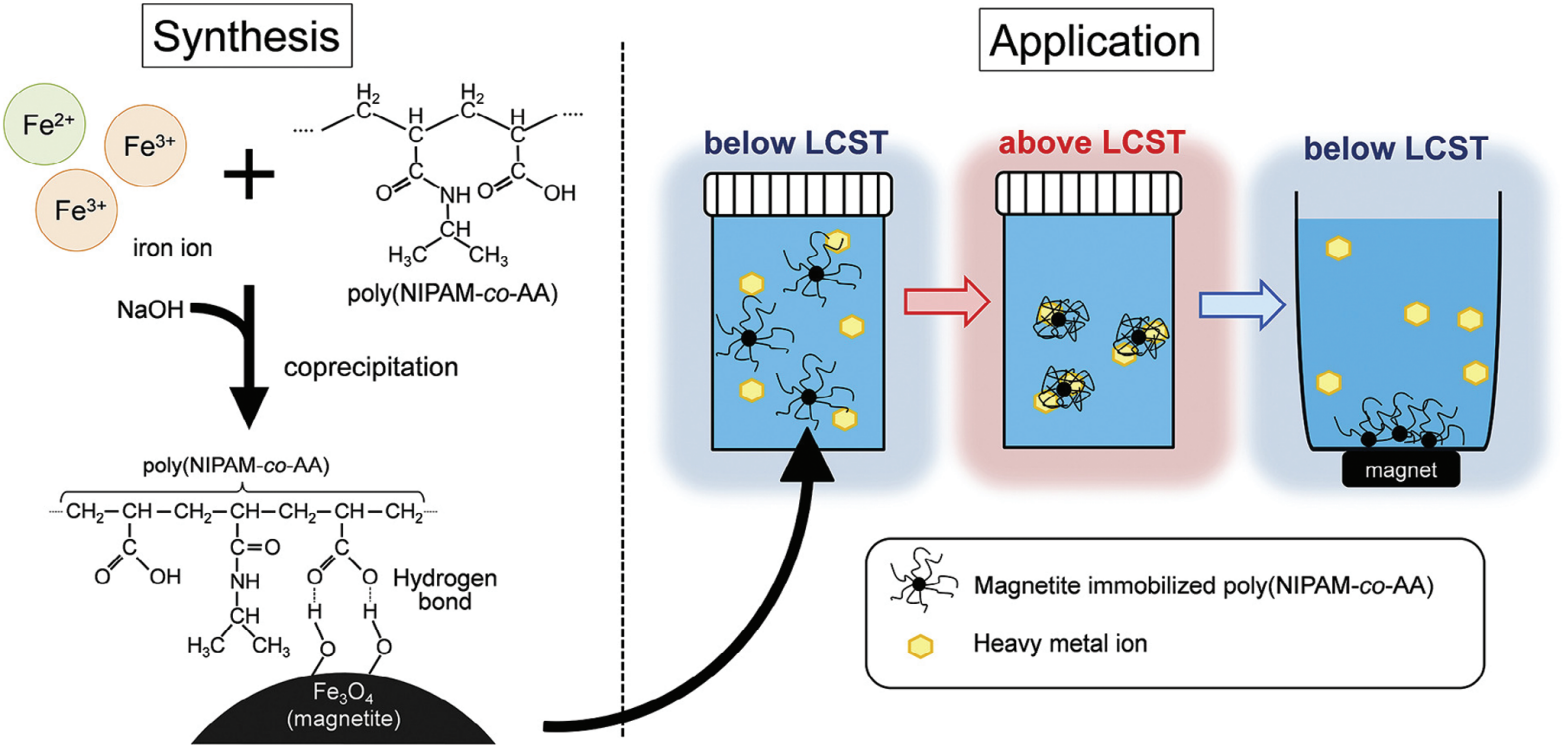
Representative image of thermo‐responsive particles developed for water treatment, illustrating the synthesis and the application of the nanosystem. Reprinted with permission from.^[^ ^143^ ^]^

Another important application of thermo‐responsive polymer is represented by purification and separation systems.^[^ ^131^, ^139^, ^140^ ^]^ These smart materials have shown significant promise due to their ability to adapt their properties in response to temperature changes, making them highly effective in various separation processes. One notable advancement involves achieving high adsorption capacities with thermo‐responsive polymers: for instance, adsorption is notably higher at LCST, where the thermo‐responsive component of the adsorbent transitions to a swollen coil conformation. This structural change increases the material's capacity to capture ions from wastewater.^[^ ^141^, ^142^ ^]^ In contrast, at temperatures below the LCST, the adsorbent undergoes de‐swelling, which facilitates the release of the adsorbed ions. In another study, Hayashi and coworkers employed a thermo‐responsive copolymer, poly(N‐isopropylacrylamide‐co‐acrylic acid), which was functionalized with magnetite nanoparticles through a co‐precipitation technique (**Figure** **7**).^[^ ^143^ ^]^ This thermo‐responsive copolymer was used as an adsorbent for copper ions in wastewater. Before utilization, the polymer was characterized using various techniques, including TEM, FT‐IR, thermogravimetric analysis and vibrating sample magnetometry.

These characterizations confirmed a high density of chelating groups in the adsorbent, which facilitated complexation and electrostatic interactions as the primary mechanisms for copper ion adsorption. The study demonstrated that adsorption and desorption could be efficiently controlled through temperature variations between 60 and 20 °C, without the need for organic solvents, making the process environmentally friendly.^[^ ^144^ ^]^

Additionally, the adsorbent could be easily recovered from the solution using a magnet and was recyclable. A specific application of separation technology can be found in membranes, which are simple systems capable of separating and purifying substances at a low operational cost and with a low environmental impact, making them very suitable for large‐scale industrial applications due to their high efficiency. They are predominantly used in the food and environmental sectors, with the purpose of extracting analytes by adsorbing them.^[^ ^145^, ^146^ ^]^ In particular, thermo‐responsive polymers can alter their physicochemical properties based on temperature changes. This allows for the modulation of membrane functionality by simply adjusting the temperature.^[^ ^147^ ^]^ The operating principle relies on the temperature‐dependent structural changes of the membrane: specifically, once the LCST is exceeded, these membranes undergo a change in nanopore size, making the membrane selective for certain substances based on their size. A notable example involves polymers with an LCST: these polymers display distinct behaviors depending on whether the temperature is above or below their LCST. Below the LCST, the polymers remain in a swollen state, causing the membrane pores to expand and facilitate the passage of analytes. In contrast, when the temperature exceeds the LCST, the polymers collapse, leading to a reduction in pore size and restricting analyte passage.^[^ ^85^ ^]^ This reversible change in pore dimensions enables effective control over the separation and extraction of various substances.^[^ ^148^ ^]^

### 6.3 Gene Delivery

Gene delivery and gene therapy represent significant advancements in the treatment of genetic disorders and infections. Gene delivery involves introducing DNA or RNA into cells to provide new genetic information, such as enabling cells to produce a protein that is missing due to a pathological condition.^[^ ^149^ ^]^ It applies genetic principles to treat diseases by introducing or modifying genetic material within a patient's cells. The goal is to correct malfunctioning genes or introduce new functions to combat diseases. Self‐assembled micelles, particularly those utilizing tri‐block or graft copolymers, are a key method in this area. These nanoparticles can encapsulate nucleic acids via electrostatic interactions, as the negatively charged nucleic acids bind to positively charged molecules.^[^ ^150^ ^]^ Despite the efficiency of viruses in gene delivery due to their natural evolution, they have limitations, including safety concerns and immune responses. To address these limitations, researchers have developed non‐viral vectors using polymers and lipids.^[^ ^151^ ^]^ These non‐viral systems can encapsulate RNA or DNA and deliver them into target cells.

Although non‐viral vectors are generally less efficient than viral systems, they offer advantages such as lower cost, better availability, reduced immune response, and fewer limitations on the size of transgenic DNA. In the context of polymeric vectors, the synthesis of polyplexes requires selecting an effective polycation. While polycations can efficiently bind to negatively charged nucleic acids, they often exhibit higher toxicity in relation to polyanions or non‐ionic polymers. This toxicity results from their tendency to trigger opsonization and interact with cellular membranes, which can complicate their application. The first significant application of thermo‐responsive polymers in gene delivery was demonstrated by Yokoyama and coworkers, who operated a combination of PNIPAM, diethylaminomethyl methacrylate (DMAEMA), and butylmethacrylate.^[^ ^152^, ^153^ ^]^ Tuning the quantities of each part, it is possible to control the polymer's hydrophobic interactions with DNA, illustrating the potential to fine‐tune polymer properties for optimized gene delivery systems through temperature‐responsive mechanisms. These polymers offer substantial advantages for gene delivery by helping the reversible compaction of DNA into nanoparticles smaller than 200 nm.^[^ ^154^ ^]^ When the temperature exceeds the LCST, these polymers exhibit increased hydrophobic interactions, thzt reduces the necessity for excessive polycation in the DNA complexes. Excessive polycation often leads to non‐specific interactions with various biological components such as plasma proteins, complement factors, fibrinogen and red blood cells.^[^ ^154^ ^]^ Such interactions can result in rapid clearance of the complexes from the bloodstream due to the formation of large polymer‐protein aggregates, which are promptly removed by phagocytic liver cells or become trapped in capillary beds. This aggregation can obstruct blood vessels, leading to severe conditions such as pulmonary embolism and polycations can also trigger the complement system. By adjusting the content of amino group‐containing units (e.g., DMAEMA) in the copolymer, ionic interactions between the copolymer and DNA can be controlled. This adjustment allows for easier dissociation of the complexes for transcription below the LCST, while above the LCST, enhanced hydrophobic interactions ensure tight complex formation.^[^ ^153^ ^]^ This temperature‐dependent behavior enables controlled gene delivery and expression, introducing a novel approach to gene expression control. Furthermore, varying the DMAEMA/NIPAM ratio in the copolymer influences the charge of the complexes, which in turn affects transfection efficiency and cytotoxicity.^[^ ^155^ ^]^

### 6.4 Imaging

After the injection, is required a tool to detect the actual biodistribution of the nanoparticles.

In this context, imaging is one of the most exploited techniques in order to find out the path of nanoparticles following injection into the bloodstream.

To ensure that nanoparticles effectively reach their planned target, such as a tumor, and to assess their interaction with the immune system, precise tracking and characterization methods are essential. Ma and coworkers have synthesized nanoparticles based on tetraphenylethylene and NIPAM, designed as lysosome‐specific fluorescent probes for bioimaging, demonstrating low cytotoxicity, high specificity and excellent resistance to photobleaching.^[^ ^156^ ^]^ One straightforward method for tracking nanoparticles is labelling them with radioactive tracers.^[^ ^157^ ^]^ This approach allows for detection using imaging techniques such as scintigraphy, which provides information on the distribution and localization of the nanoparticles within the body. Additionally, recent advancements in imaging have significantly benefited from the development of quantum dots, representing cutting‐edge nanotechnology that offers superior imaging capabilities compared to traditional organic dyes. The stability of quantum dots is attributed to their inorganic nature, which prevents the rapid degradation seen with organic dyes. Nanoscale quantum dots, typically ranging from 2 to 50 nm in diameter, emit light that changes from blue to red as their size increases. This size‐dependent emission allows for multiplexing in imaging studies, where multiple quantum dots with different emission wavelengths can be used simultaneously to track various nanoparticles or biological processes.^[^ ^158^ ^]^ Quantum dots photostability further enhances their utility in vivo imaging studies as they can remain fluorescent for months, providing continuous and reliable tracking of nanoparticles over extended periods. In particular, it has developed and produced a new class of thermo‐responsive semiconducting polymer nanoparticles designed for high‐contrast photoacoustic imaging of tumors. These advanced agents are built from semiconducting polymer brushes featuring poly(N,N‐dimethylacrylamide)‐r‐(hydroxypropyl acrylate) (PDMA‐r‐HPA) grafts.^[^ ^159^ ^]^ The PDMA‐r‐HPA polymers exhibit thermo‐responsive properties, undergoing a reversible phase transition in response to temperature changes. This phase transition is crucial for the performance of the PA imaging agents. This polymer, specifically called SPNph1 and SPNph2,^[^ ^160^ ^]^ presents an LCST: above its value, these nanoparticles undergo phase separation, leading to their aggregation. This aggregation increases their size, enhancing heat dissipation and thus amplifying the PA signal. The enhanced PA signals can be achieved through thermal stimulation, which remotely improves the signal‐to‐background ratio (SBR) in PA imaging.^[^ ^161^ ^]^ To explore the thermo‐responsive PA amplification, an 808 nm near‐infrared laser was used to heat the tumor tissues. This temperature increase, which lasted for just one min at 48 °C, was found to be non‐damaging. Without laser irradiation, both SPNph1 and SPNph2 exhibited relatively low PA signals. However, after 1 min of laser exposure, the PA signal of tumors treated with SPNph1 significantly increased.^[^ ^161^ ^]^

**Figure 8:**
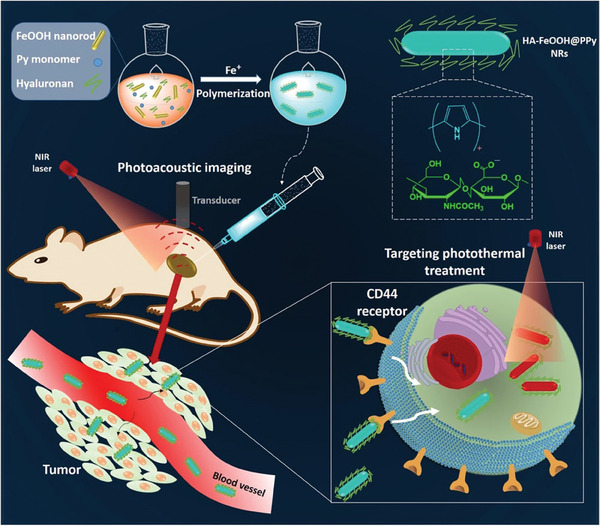
Schematic representation for the synthesis and the applications of nanoagents based on hyaluronan (HA)‐coated FeOOH@polypyrrole (FeOOH@PPy) nanorods (HA‐FeOOH@PPy NRs) for photoacoustic imaging (PAI)‐guided photothermal therapy. Reprinted with permission from.^[^ ^162^ ^]^

The PDMA‐r‐HPA grafted semi conducting NPs, particularly SPNph1, demonstrated an increase in PA signal when the temperature was raised above the LCST. This performance underscores the advantages of using thermo‐responsive polymers in PA imaging. The ability to remotely control PA signals via laser‐induced heating enhances the potential for these agents in sensitive and precise tumor imaging.^[^ ^161^ ^]^ In another study, rod‐shaped nanoparticles based on hyaluronan were developed and functionalized with tumor‐targeting ligands to enhance accumulation and achieve uniform distribution across tumor tissues (**Figure** **8**).^[^ ^162^ ^]^ These nano‐systems demonstrated excellent biocompatibility and physiological stability in various media. Notably, these nanorods exhibited strong near‐infrared absorbance, high photothermal conversion efficiency, and remarkable stability under repeated laser irradiation. In addition to their photothermal properties, they proved to be highly effective as contrast agents for photoacoustic imaging, enabling precise localization of cancerous tissues and accurate guidance for photothermal therapy. Both in vitro and in vivo studies confirmed the significant photothermal anticancer efficacy of these nanoparticles. Tumor‐bearing mice treated with photothermal therapy fully recovered within 17 days, demonstrating complete tumor ablation with no apparent side effects.

## 7 Conclusion

Thermo‐responsive polymers have emerged as a cutting‐edge tool in nanomedicine, paving the way for innovative approaches to targeted drug delivery and advanced therapeutic strategies. The use of external stimuli allows for non‐invasive drug administration and localized distribution, minimizing the damage to healthy tissue. Although the results are highly promising, significant challenges remain before these systems can advance to clinical application. The clinical translation of thermo‐responsive polymers faces multiple hurdles. Ensuring their biocompatibility and addressing potential toxicity iscritical, as both the polymers and their degradation products must be proven to be safe for long‐term use. Additionally, the immune response poses a significant challenge. The introduction of nanoparticles into the body can trigger immune activation, leading to rapid clearance from circulation, inflammation, or adverse reactions, thereby reducing therapeutic efficacy. Another critical aspect in the clinical phase is related to their biodistribution and targeting since possible off‐target accumulation can lead to unintended toxicity. The dynamic and complex physiological environment can also impact the stability and responsiveness of the nanoparticles, potentially causing premature drug release or a diminished response to thermal stimuli. Furthermore, variability in patient‐specific factors such as body temperature, metabolism, and disease state can affect their performance, complicating dose optimization and predictability. The complexity of producing nanoparticles with consistent quality on a large scale further complicates their development. Storage and stability also present significant obstacles, as maintaining the structural integrity and bioactivity of the nanoparticles requires advanced solutions to prevent premature degradation and drug loss. Effective sterilization methods must also be developed that do not compromise the functionality or structure of these systems. Regulatory hurdles represent another major challenge. Nanoparticles used for drug delivery are classified as combination products, requiring stringent evaluation of both the carrier and the therapeutic agent, often leading to longer approval timelines compared to conventional drugs. In addition, manufacturing processes and raw materials must ensure sterility, adding complexity to regulatory compliance. High costs and limited patent protection further hinder the commercial viability of these systems. To address these challenges, several strategies can be adopted. Ensuring biocompatibility and minimizing toxicity requires the design of polymers using FDA‐approved materials, such as PEG or natural polymers. Functionalizing these materials with non‐immunogenic groups can further improve their compatibility. Additionally, engineering polymers with predictable degradation pathways, producing safe and biologically inert by‐products, is essential.

Comprehensive testing, both in vitro and in vivo, should be employed to assess long‐term safety, focusing on systemic toxicity, chronic inflammation, and bioaccumulation. Mitigating immune responses is another crucial step. Surface modifications, such as PEGylation or the use of zwitterionic polymers, can help nanoparticles evade immune detection and extend their circulation time. To improve biodistribution and targeting, nanoparticles can be functionalized with targeting ligands like folic acid or peptides to ensure accumulation at specific sites. The thermal sensitivity of these systems can be optimized by fine‐tuning their transition temperatures to align with physiological conditions in the target tissue. Developing computational models that predict nanoparticle behavior based on patient‐specific parameters, such as metabolism or body temperature, can aid in customizing treatments and improving their efficacy. The production and scalability of these nanoparticles also demand attention. Advanced techniques, such as microfluidic synthesis, allow for the production of nanoparticles with high encapsulation efficiency and uniform size distribution. Additionally, adopting continuous flow reactors or other scalable systems can help achieve consistent quality in large‐scale production. Stabilizers or cross‐linkers could be used during storage and transport. To solve sterilization challenges could be involved the use of non‐invasive sterilization techniques like gamma irradiation or filtration, which preserve the functional and structural integrity of the nanoparticles. Cryopreservation can prevent aggregation and degradation during storage. Furthermore, developing smart packaging solutions that control humidity and temperature can extend the shelf life of these materials. Regulatory hurdles can be navigated through early engagement with regulatory agencies to align on safety and efficacy standards. Developing standardized assays for nanoparticle evaluation and equipping multidisciplinary teams with regulatory expertise can streamline the approval process. Broader patent claims covering both the polymer formulation and unique applications may enhance intellectual property protection.

Finally, the design of clinical trials should consider patient‐specific factors. Stratifying patients based on disease state, metabolism, and body temperature can improve predictive accuracy. Employing adaptive trial designs, which allow for dynamic adjustments in dosing and protocols based on interim results, can further enhance trial efficiency and success rates. In summary, while thermo‐responsive polymers hold great promise for nanomedicine studies overcoming challenges related to their clinical translation, in vivo performance and regulatory, their approval will be essential to unlock their potential in advanced therapeutic applications. By integrating these strategies, the transition from laboratory to clinic can be accelerated, paving the way for innovative, effective, and safe therapeutic solutions.

## Conflict of Interest

The authors declare no conflict of interest.
